# Prevention of unwanted recombination at damaged replication forks

**DOI:** 10.1007/s00294-020-01095-7

**Published:** 2020-07-15

**Authors:** Carl P. Lehmann, Alberto Jiménez-Martín, Dana Branzei, José Antonio Tercero

**Affiliations:** 1 Centro de Biología Molecular Severo Ochoa (CSIC/UAM), Cantoblanco, 28049 Madrid, Spain; 2 Present Address: Centro Andaluz de Biología del Desarrollo (CSIC/UPO), 41013 Seville, Spain; 3 IFOM, The FIRC Institute of Molecular Oncology, Via Adamello 16, 20139 Milan, Italy; 4 Istituto di Genetica Molecolare, Consiglio Nazionale delle Ricerche (IGM-CNR), Via Abbiategrasso 207, 27100 Pavia, Italy

**Keywords:** DNA recombination, DNA replication forks, DNA damage bypass, Template switching, Mgs1, Genome stability

## Abstract

Homologous recombination is essential for the maintenance of genome integrity but must be strictly controlled to avoid dangerous outcomes that produce the opposite effect, genomic instability. During unperturbed chromosome replication, recombination is globally inhibited at ongoing DNA replication forks, which helps to prevent deleterious genomic rearrangements. This inhibition is carried out by Srs2, a helicase that binds to SUMOylated PCNA and has an anti-recombinogenic function at replication forks. However, at damaged stalled forks, Srs2 is counteracted and DNA lesion bypass can be achieved by recombination-mediated template switching. In budding yeast, template switching is dependent on Rad5. In the absence of this protein, replication forks stall in the presence of DNA lesions and cells die. Recently, we showed that in cells lacking Rad5 that are exposed to DNA damage or replicative stress, elimination of the conserved Mgs1/WRNIP1 ATPase allows an alternative mode of DNA damage bypass that is driven by recombination and facilitates completion of chromosome replication and cell viability. We have proposed that Mgs1 is important to prevent a potentially harmful salvage pathway of recombination at damaged stalled forks. In this review, we summarize our current understanding of how unwanted recombination is prevented at damaged stalled replication forks.

## Introduction

In every cell cycle, cells are faced with the challenging task of preserving the integrity of the genome while making an accurate copy of it. Failures in this process can cause cell death or different pathologies that in metazoans can lead to cancer or developmental abnormalities, among others (Aguilera and Garcia-Muse 2013; Zeman and Cimprich 2014; Cortez 2019). The complexity of replicating entire genomes requires efficient replication machinery as well as multiple regulatory and control mechanisms. During chromosome replication, the DNA is unpacked and unwound, which leaves it highly exposed to different types of insults, putting genome integrity at risk (Aguilera and Garcia-Muse 2013; Zeman and Cimprich 2014; Cortez 2019). A main source of genomic instability is the inevitable presence of DNA lesions that frequently cause fork stalling. To ensure genome stability and faithful completion of DNA replication under these conditions, replication forks have to be protected to avoid collapse, and the different DNA lesions must be either repaired or tolerated (Branzei and Foiani 2010; Branzei and Psakhye 2016; Singh and Wu 2019).

Homologous recombination plays an important role in the maintenance of genome integrity, particularly relevant in coping with double-stranded DNA breaks (Jasin and Rothstein 2013; Wright et al. 2018). However, unscheduled recombination during chromosome replication is potentially dangerous as it can lead to deleterious genomic rearrangements and faulty replication (Branzei and Szakal 2017; Carr and Lambert 2013). This problem is minimized by global inhibition of recombination at replication forks, which in budding yeast is largely driven by recruitment of the Srs2 helicase to SUMOylated PCNA (Motegi et al. 2006; Papouli et al. 2005; Pfander et al. 2005). Yet, in the case of template switch recombination, Srs2 is locally counteracted at sites of perturbed replication to allow this error-free mode of DNA damage bypass (Urulangodi et al., 2015). Recently, our work has shown that the evolutionarily conserved Mgs1/WRNIP1 ATPase is important to prevent a salvage pathway of recombination at damaged and stalled DNA replication forks (Jiménez-Martín et al. 2020), uncovering a new role for this protein in the intricate network of mechanisms that contribute to maintaining genome stability.

## SUMO-PCNA and Srs2 mediated global inhibition of recombination at DNA replication forks

**Fig. 1:**
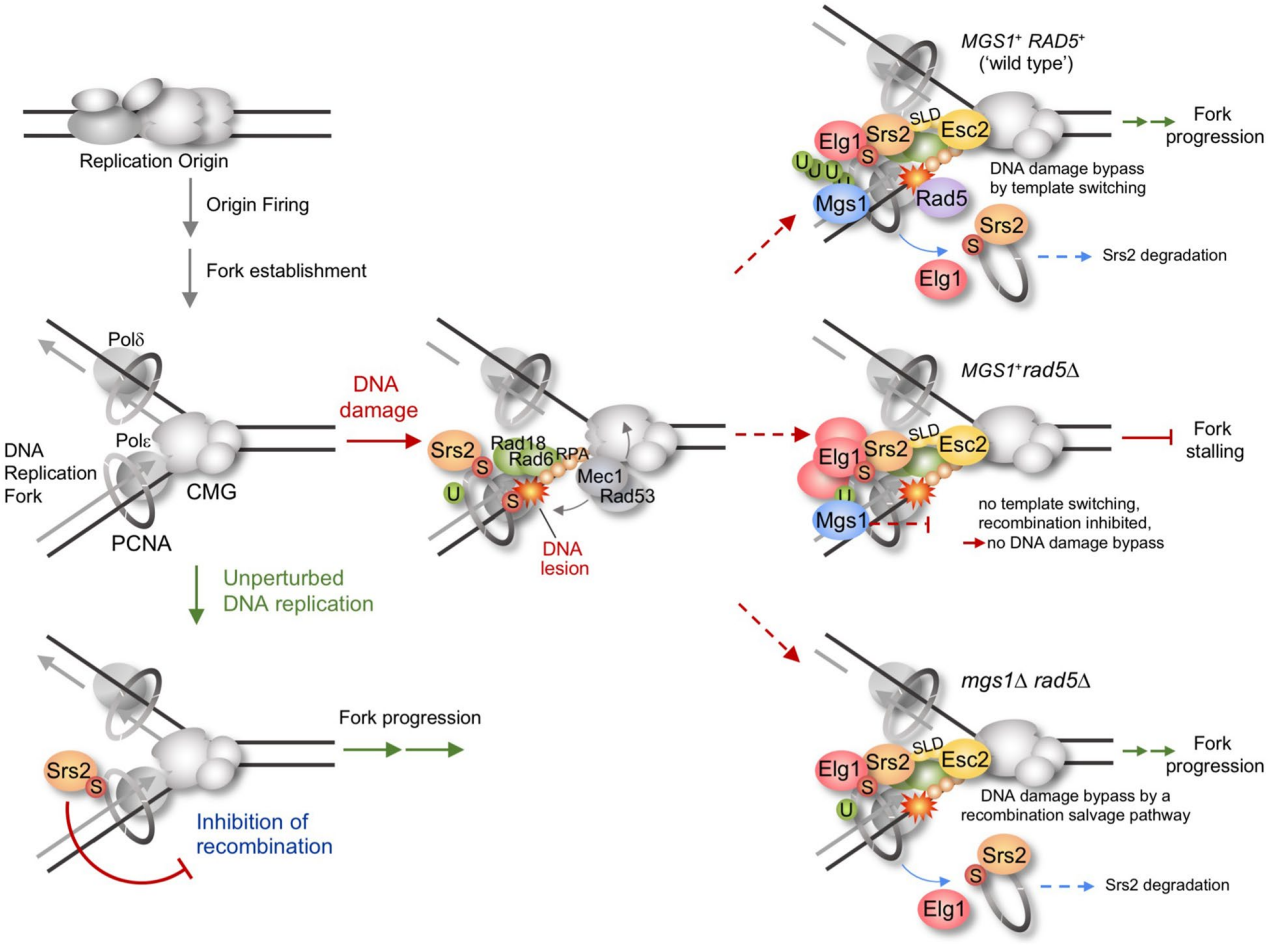
Schematic representation of the control of recombination at DNA replication forks during unperturbed replication and in the face of DNA damage. In the absence of DNA lesions, Srs2 inhibits homologous recombination at ongoing replication forks. At damaged stalled forks, ‘wild-type’ cells counteract Srs2 and bypass DNA lesions by error-free recombination-mediated template switching. In cells lacking Rad5, forks block due to the existence of DNA lesions, since there is no template switching and an alternative recombination bypass pathway is prevented by the presence of Mgs1. In the absence of both Mgs1 and Rad5, a recombination salvage pathway is allowed and drives DNA damage bypass. See details in the text

During chromosome replication, unchecked recombination events can be detrimental for cells, as they can cause undesirable genome rearrangements and accumulation of DNA intermediates that overwhelm the resolution activities cells are endowed with (Branzei and Szakal 2017). To avoid these dangerous situations for genome stability, eukaryotic cells have mechanisms that prevent unscheduled replication-associated recombination during unperturbed genome duplication. The best-known mechanism in this regard is mediated by the interaction of the Srs2 helicase with SUMOylated PCNA. During chromosome replication, Ubc9 SUMO conjugating enzyme and Siz1 SUMO ligase modify PCNA, the processivity factor for replicative DNA polymerases, by binding the small molecule SUMO to its conserved residue K164 and, to a lesser extent, K127 (Hoege et al. 2002). PCNA SUMOylation leads to the recruitment of the anti-recombinogenic helicase Srs2, which prevents unscheduled recombination at ongoing replication forks (Motegi et al. 2006; Papouli et al. 2005; Pfander et al. 2005) (Fig. 1). This inhibition involves disruption of Rad51 presynaptic filaments (Krejci et al. 2003; Veaute et al. 2003) and inhibition of Rad52 (Arbel et al. 2020a, b; De Tullio et al. 2017). Arbel et al. (2020a) showed that overexpression of Rad52 or of the PCNA-unloader Elg1 bypasses the anti-recombination function of Srs2, providing greater understanding about the mechanism mediated by this protein. PCNA SUMOylation at K164 is evolutionarily conserved (Gali et al. 2012; Moldovan et al. 2012) and there are functional homologues of Srs2 in other organisms. In mammalian cells, SUMOylated-PCNA enhances its interaction with PARI. Like Srs2, PARI plays an antirecombinogenic role by interfering with the formation of RAD51-DNA structures and is important to prevent inappropriate recombination at replication forks (Burkovics et al. 2016; Moldovan et al. 2012). However, given that PCNA SUMOylation in mammals is less prominent than in yeast, it is possible that other functional Srs2 homologues exist and their recruitment may be different from that of Srs2. In this vein, two human RecQ helicases, RECQL5 and BLM, are known to suppress homologous recombination. In the case of BLM, its association to replication forks is important for its anti-recombination function (reviewed in Branzei and Szakal 2017).

## DNA damage tolerance mechanisms

While general prevention of recombination events is essential for maintaining genome stability during unperturbed chromosome replication, the situation becomes more complex in the face of DNA damage, as template switching—a recombination-mediated mechanism—is necessary to tolerate DNA lesions in S-phase (Branzei and Psakhye 2016; Saugar et al. 2014). Unrepaired DNA lesions during replication are a serious threat to cells, as they may impede fork progression. Extended fork stalling can result in DNA breakage as a consequence of the fragility of single-stranded DNA (ssDNA). Therefore, prolonged stalling must be avoided to prevent this important cause of genomic instability and to ensure the completion of chromosome replication. To circumvent these problems, cells use DNA damage tolerance pathways that allow the bypass of fork blocking lesions, postponing their repair for later in the cell cycle (Branzei and Psakhye 2016; Saugar et al. 2014).

When a replication fork encounters a DNA lesion that blocks its progression, the replicative helicase and the DNA polymerases may uncouple, originating a long area of ssDNA that is covered by RPA (replication protein A) (Fig. 1). This coated ssDNA is the signal for the activation of the S-phase checkpoint (Zou and Elledge 2003), a surveillance pathway required to preserve the integrity of damaged and stalled forks (Lopes et al. 2001; Tercero and Diffley 2001; Tercero et al. 2003). RPA-coated ssDNA also triggers the binding of the E3-ubiquitin ligase Rad18 to chromatin, which recruits the E2-conjugating protein Rad6 (Davies et al. 2008) to initiate DNA damage tolerance mechanisms (Fig. 1). Rad18 and Rad6 form a heterodimer that modifies PCNA by monoubiquitylation at the same K164 residue at which this sliding clamp is SUMOylated (Hoege et al. 2002). PCNA is composed of three identical subunits that encircle the DNA and, at least in budding yeast, SUMOylated PCNA is likely the physiological substrate of Rad18 (Parker and Ulrich 2012).

PCNA monoubiquitylation has been found in all eukaryotes and activates translesion DNA synthesis (TLS) (Stelter and Ulrich 2003), a direct mechanism of DNA damage bypass. TLS is carried out by specialized low-fidelity polymerases that replace the stalled replicative polymerases and are able to replicate across the lesions in a frequently error-prone process (Sale 2013). Monoubiquitylated PCNA can be further polyubiquitylated (Fig. 1) by extension of the previously bound ubiquitin to K63-linked chains (Hoege et al. 2002). In budding yeast, PCNA-polyubiquitylation is conducted by the E3-ubiquitin-ligase Rad5 (HLTF/SHPRH in mammalian cells), which also has DNA-dependent ATPase/helicase activity, in conjunction with the E2-conjugating complex Ubc13-Mms2 (mammalian UBC13-UEV1) (Hoege et al. 2002). By means of a not yet fully understood mechanism, the polyubiquitylation of PCNA promotes another type of DNA damage bypass that is mediated by transient template switch recombination, which requires Rad5-helicase activity and is error-free (Branzei and Psakhye 2016; Branzei and Szakal 2016; Saugar et al. 2014). In this mode of damage bypass, the nascent DNA strand blocked by the lesion uses the newly synthesized strand of the undamaged sister chromatid as a template for replication across the lesion, a recombination process that can be visualized as the formation of X-shaped sister chromatid junctions (SCJs) (Branzei et al. 2008; Giannattasio et al. 2014; Zhang and Lawrence 2005). Although TLS and template switch recombination are different modes of tolerating DNA lesions, they are interconnected through Rad5 (Pages et al. 2008; Fan et al. 2018; Gallo et al. 2019; Kuang et al. 2013), which may be considered a central protein in DNA damage tolerance.

## Allowing recombination by template switching at damaged replication forks

Error-free template switch recombination is fundamental for coping with DNA damage and, at least in response to several types of DNA lesions, is the favored mode of DNA damage bypass during replication (Gonzalez-Huici et al. 2014; Ortiz-Bazán et al. 2014). Given that, as previously explained, recombination is globally inhibited at replication forks by the antirecombinase Srs2, an important question to address was how DNA damage bypass by template switching, which depends on recombination factors (Branzei et al. 2008; Vanoli et al. 2010), is, however, allowed at damaged stalled forks. Urulangodi et al. (2015) resolved this problem by showing that template switch recombination is enabled at sites of compromised DNA replication, where the Srs2 helicase is locally counteracted (Fig. 1). They found that the conserved SUMO-like domains (SLDs)-containing protein Esc2 plays a key role in limiting local levels of Srs2 by restricting its recruitment to chromatin and promoting its turnover. These reduced levels of Srs2 allow the formation of Rad51 filaments locally, at damaged forks, which in turn facilitates the bypass of DNA damage by template switch recombination. Urulangodi et al. (2015) showed that Esc2 preferentially binds structures that originate at damaged and stalled replication forks. In this context, Esc2 facilitates stable binding of the PCNA-unloader Elg1 to damaged forks, which contributes to local unloading of SUMO-PCNA, together with bound Srs2 (Fig. 1). Moreover, Esc2 interacts via its SLDs with the SUMO-interacting motifs (SIMs) of Srs2 and Slx5, and these interactions locally channel Srs2 for proteasome-dependent degradation mediated by the SUMO-targeted ubiquitin ligase (STUbL) complex Slx5/Slx8 (Urulangodi et al. 2015). Thus, both increased turnover of the Srs2 anti-recombinase and Elg1-dependent local unloading of SUMO-PCNA restrict the levels of Srs2 at sites of perturbed replication, which facilitates template switching recombination at stalled forks while recombination is still globally restricted at undamaged ongoing replication forks.

## Mgs1-dependent inhibition of a salvage pathway of recombination at damaged replication forks

As explained above, down-regulation of Srs2 at damaged stalled forks allows the bypass of DNA lesions by template switching (Urulangodi et al. 2015). These findings, in turn, raise the question of how template switch-mediated recombination is specifically facilitated but, in spite of Srs2 antirecombinase being counteracted, other modes of recombination that could be potentially dangerous for genome stability are inhibited at damaged forks. We addressed this question and found that Mgs1, an evolutionarily conserved AAA + ATPase (WRNIP1 in mammalian cells, RarA/MgsA in bacteria) (Hishida et al., 2001), contributes to preventing unwanted recombination at damaged and stalled replication forks (Jiménez-Martín et al. 2020).

Cells lacking the central DNA damage tolerance protein Rad5 are extremely sensitive to agents that cause DNA lesions or replicative stress, and cannot complete chromosome replication in the presence of DNA lesions that block fork progression, such as those caused by the alkylating agent methyl methanesulfonate (MMS) (Karras and Jentsch 2010; Minca and Kowalski 2010; Ortiz-Bazán et al. 2014). We found that elimination of Mgs1 or its ATPase activity suppresses the high sensitivity of Rad5-deficient cells to MMS or hydroxyurea (HU). A likely explanation for this suppression is the observation that in cells lacking Rad5, the absence of Mgs1 facilitates an alternative pathway of DNA damage bypass that allows overcoming of MMS-induced DNA lesions and the completion of chromosome replication (Jiménez-Martín et al. 2020) (Fig. 1). This pathway is driven by homologous recombination, as shown by genetic and 2D-gel analyses, and depends on at least the recombination proteins Rad52 and Rad59. It also requires the function of the polymerase δ and PCNA modification at the K164 residue. Since in the absence of Rad5 there is neither template switch recombination nor PCNA polyubiquitylation, the mode of recombination that the lack of Mgs1 promotes in this context can be considered a salvage pathway. This type of recombination is potentially toxic because it can cause genomic rearrangements and so is usually restricted to late S-phase or G2/M (Branzei and Szakal 2016; Prado 2018). The absence of Srs2 also allows a recombination salvage pathway that, like the one described above, is Rad52 and Rad59-dependent (Arbel et al. 2020a, b). Therefore, it is possible that Srs2 and Mgs1 are inhibiting the same type of salvage pathway.

As with template switching at damaged stalled forks (Urulangodi et al. 2015), Esc2 and Elg1 are necessary for the recombination salvage pathway that the absence of Mgs1 allows in cells lacking Rad5 (Jiménez-Martín et al. 2020). These common requirements strongly suggest that both pathways are mediated by a similar mechanism that is operated by Esc2 and Elg1 (Fig. 1). Thus, in cells lacking Rad5 and Mgs1, Esc2 would bind to damaged replication forks, facilitating enhanced association of Elg1 to these sites of perturbed replication. This would lead to the unloading of the Srs2 antirecombinase bound to SUMO-PCNA and concomitant or subsequent proteasome-dependent degradation mediated by Slx5/Slx8. Local Srs2 degradation would facilitate the binding of Rad51 to damaged forks, followed by a recombination-driven bypass pathway that is dependent on Rad52/Rad59 and Pol δ (Jiménez-Martín et al. 2020). All this described recombination-driven replication process is prevented in the presence of Mgs1, explaining why in *MGS1*^+^*rad5*Δ cells, where there is neither template switching nor an alternative mode of recombination-mediated DNA damage bypass, replication forks block in the face of DNA lesions (Ortiz-Bazán et al. 2014) (Fig. 1). Likewise, our data may explain why mitotic recombination is increased in a *mgs1*Δ mutant (Branzei et al. 2002; Hishida et al. 2001). These results may also help to understand why in ‘wild-type’ (*MGS1*^+^*RAD5*^+^) cells Rad5-dependent template switch recombination is fully operative for DNA damage bypass, whereas potentially dangerous salvage recombination is inhibited at damaged forks despite the local reduction of Srs2 levels. This inhibition, in turn, could help to channel DNA damage bypass to error-free template switch recombination.

Previous work showed that Mgs1 interacts with PCNA, preferentially with polyubiquitylated PCNA via its ubiquitin-binding zinc-finger (UBZ) domain, which facilitates its recruitment to replication stress sites (Saugar et al. 2012). Moreover, it is known that Mgs1 has ssDNA annealing activity and interacts with polymerase δ (Hishida et al. 2001, 2002, 2006; Branzei et al. 2002; Vijeh Motlagh et al. 2006). In cells lacking Rad5, we found a correlation between increased levels of Elg1 at forks and the presence of Mgs1 (Jiménez-Martín et al. 2020). Taking all this information into account, future work will be necessary to elucidate, at the molecular level, how exactly Mgs1 exerts its action in preventing recombination and how this is coordinated with the DNA damage tolerance proteins and in particular with Rad5. In any case, the data strongly suggest that Mgs1 is a key piece within the mechanisms that prevent unwanted recombination at damaged replication forks. We believe these new findings provide insights into how unwanted recombination is restricted at forks while DNA damage bypass by template switching is favored to facilitate completion of chromosome replication and genome stability maintenance.
